# Rapid and Sensitive Detection of Pentachloronitrobenzene by Surface-Enhanced Raman Spectroscopy Combined with Molecularly Imprinted Polymers

**DOI:** 10.3390/bios12020052

**Published:** 2022-01-19

**Authors:** Jing Neng, Caiping Liao, Yazhi Wang, Yan Wang, Kai Yang

**Affiliations:** College of Food Science and Technology, Zhejiang University of Technology, Hangzhou 310014, China; nengjing@zjut.edu.cn (J.N.); 13628612080@163.com (C.L.); wyzzjut@163.com (Y.W.); wangyan062006@zjut.edu.cn (Y.W.)

**Keywords:** surface-enhanced Raman spectroscopy, molecularly imprinted polymers, detection, pentachloronitrobenzene, oil-soluble silver nanoparticles, pesticide residues

## Abstract

Molecularly imprinted polymers (MIPs) specifically targeting pentachloronitrobenzene (PCNB) and containing silver nanoparticles have been prepared by free radical polymerization reaction using methyl methacrylate (MMA) as a functional monomer, PCNB as a template molecule, 1,4-butanedioldimethacrylate as a cross linker, lauroyl peroxide (LPO) as an initiator, and the silver nanoparticles with the best surface-enhanced Raman scattering (SERS) effect as SERS enhancement materials. Our results indicated that MIPs specifically recognize PCNB from complex matrices. The intensity of the PCNB characteristic peak was proportional to the concentration, with a linear range of 0.005 to 0.15 μg/mL and a limit of detection of 5.0 ng/mL. The recovery rates and relative standard deviation for the detection of PCNB spiked in the rice samples were from 94.4% to 103.3% and from 4.6% to 7.4%, respectively. The experimental results are consistent with those by the GC-MS method, indicating that the rapid detection of PCNB in food matrices by SERS-MIPs is reliable. In view of the insolubility of PCNB in water, oil-soluble silver nanoparticles were synthesized which can be expanded to detect oil-soluble toxic substances. For the first time, the proposed method provides a point-of-care and cost-effective tool for rapidly detecting PCNB in food matrices with high sensitivity and selectivity by employing SERS-MIPs method.

## 1. Introduction

Pesticides play an important role in preventing crop diseases and pests and in maintaining agricultural production. However, the excessive use of pesticides will inevitably make the pesticide residues exceed the national standards in vegetables, fruits, and other agricultural products, which will lead to the decline in food quality and harm the health of people after ingestion.

Pentachloronitrobenzene (PCNB) is an oil-soluble fungicide belonging to organochlorine substituted benzene. The main mechanism of action of PCNB is to prevent and control plant pathogens by interfering with cell mitosis and inhibiting spore formation. It mainly uses seed coating or seed dressing methods to control cotton seedling diseases, blight disease, wheat, sorghum smut, gold worm, or soil treatment methods to control eggplant blight, cataplash disease, and watermelon blight control by irrigation. Due to its high efficiency, low cost, and long-lasting effects, it has been widely used in the control of many kinds of seed diseases and pests [1].

However, the gradual accumulation of PCNB residues in human body may cause liver damage, endocrine disorders, reproductive disorders, and other issues [2,3], which seriously affects human health and the survival of future generations. Therefore, the United States [4], EU (European Union) [5], and many other countries have enacted legislation to strictly control the maximum limit and use range of PCNB. The maximum residue limit for PCNB is 0.01 to 0.02 mg/kg in cereals in China according to GB 2763-2019 [6] and the maximum residues of PCB for vegetables, fruits and oils were 0.05 mg/kg to 0.2 mg/kg, 0.02 mg/kg to 0.5 mg/kg, and 0.01 mg/kg to 0.5 mg/kg, respectively. However, illegal traders still overuse PCNB in the production of food raw materials. Therefore, in order to strictly control the use of PCNB, it is necessary to develop a highly sensitive, onsite, rapid and accurate PCNB detection method in food matrix.

At present, the main detection methods of PCNB are gas chromatography (GC) [7,8] gas chromatography-mass spectrometry (GC-MS) [9,10] gas chromatography-tandem mass spectrometry (GC-MS/MS) [11,12] and high-performance liquid chromatography (HPLC) [13,14]. The national standard for detection of PCNB calls for the employment of gas chromatography-mass spectrometry. Though these methods have advantages of high sensitivity and accuracy, they are time-consuming and require tedious sample pretreatment, which makes it difficult to achieve rapid, onsite detection and large-scale sample screening. Therefore, it is of great importance to develop an ultrahigh sensitive, onsite and rapid PCNB detection method for monitoring and control of the pesticide residue in food.

Surface-enhanced Raman scattering is a Raman scattering phenomenon in the presence of a nanostructured metallic surface which produces an enormous enhancement of the Raman signal. The phenomenon of SERS is explained by two mechanisms: (1) an electromagnetic enhancement mechanism associated with the surface electron movement in the metal substrate; and (2) a chemical enhancement mechanism related to charge transfer between the analyte molecules and the metal substrate. Surface-enhanced Raman spectroscopy (SERS) has remarkable advantages such as rapidity, sensitivity, non-destructive features, less sample preparation, and water-noninterference. It has been extensively applied in detection of food additives [15,16], pathogenic microorganisms [17,18], biotoxins [19,20], pesticide [21], veterinary drug residues [22,23] and environmental pollutants [24]. SERS detection is highly sensitive, which is one of the most sensitive detection techniques known at present. SERS for Rhodamine 6G has reached the limit of single molecule detection [25], and antibodies for West Nile Virus (WNV) has reached the detection limit of 50 pg/mL [26]. Under ideal condition, it can realize single molecule detection and is expected to become a quantitative tool for trace detection. However, in many cases, peaks of interfering substances may cover the target signal, resulting in false negative reading or failure to detect the target substance. On the other hand, materials with similar chemical composition or the same chemical bond would produce interference and enhance SERS signal of the target substance, thus leading to false positive results. The unique advantages of SERS technology make it play an important role in the field of ultra-sensitive and point-of-care detection. However, the detection environment in the food matrix is complex, where the unwanted components may interfere with the detection of SERS [27]. Therefore, it is necessary to combine SERS with other technologies to improve the specificity and accuracy of the detection.

Molecular imprinting technology (MIT) is a technique developed to simulate the specificity between enzymes and substrate or antigen and antibodies. Molecular imprinted polymers (MIPs) prepared by this technique are specific for the target analyte molecules. In general, the preparation of MIPs requires single-template imprints (molecularly targeted molecules or similar molecules) that can bind to functional groups (via non covalent interaction or covalent bonds) on functional monomers and cross-linking agents. The template molecule can then be removed from the prepared polymer, leaving imprinted cavities for specific rebinding of the target molecule. If the target analyte is present in the sample, MIPs can selectively absorb the target molecule into the cavity for further elution and collection. Due to their selectivity, simple preparation, low cost and long service life, MIPs have been widely used in biosensors, solid phase extraction, chromatographic separation and other related fields [28]. It also has good application prospects in natural medicine, biological engineering, and the food industry. Karimi et al. synthesized MIPs containing Fe_3_O_4_ magnetic nanoparticles, allowing it to selectively extract sulfonyamide from chicken samples [29]. A novel molecular imprinting sensor for measuring chloramphenicol(CAP) in milk samples was prepared by Alizadeh et al. [30]. Hu et al. conjugated MIPs with SERS active substrate to detect melamine in milk, and the experimental results showed that the limit of detection (LOD) and limit of quantification (LOQ) of melamine were 0.0165 and 0.055 mmol/L, respectively [31]. It is shown that the method can efficiently and quickly extract target material from samples for rapid and sensitive detection.

Silver nanoparticles (Ag NPs) were synthesized by classical hydrothermal method [32] but the silver sol was hydrophilic and difficult to dissolve with oil-soluble toxic and harmful substances in food (such as aflatoxin in moldy food, benzo pyrene in barbecue food, dimethoate in vegetables and fruits, PCNB and other pesticide residues), leading to difficulties in SERS detection. Therefore, it is necessary to synthesize oil-soluble silver nanoparticles to detect oil-soluble toxic substances.

**Figure 1:**
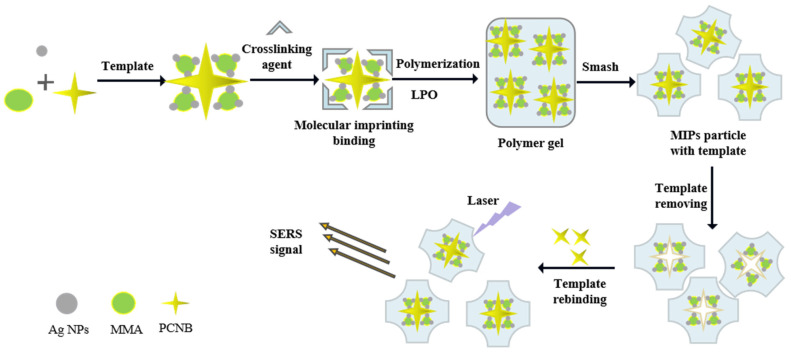
Schematic illustration of the preparation and recognition of MIPs and its application in SERS detection of PCNB.

The main purpose of this study was to combine SERS technology with MIPs (SERS-MIPs) to achieve ideal detection sensitivity and selectivity. In view of the insolubility of PCNB in water, MIPs was prepared as the SERS substrate which was embedded with oil-soluble silver nanoparticles and can specifically recognize PCNB as shown in Figure 1. MIPs specifically targeting PCNB and containing silver nanoparticles have been prepared by free radical polymerization reaction using methyl methacrylate (MMA) as a functional monomer, PCNB as a template molecule, 1,4-butanedioldimethacrylate as a cross linker, LPO as an initiator, and the silver nanoparticles with best SERS enhancement effect as SERS enhancement materials. After polymerization, the PCNB template was washed out and the MIPs was dried and crushed. The dried MIPs could specifically recognize and absorb PCNB from complex food matrix and can be reused. Finally, PCNB in MIPs was quantitatively detected by portable SERS instrument. The synthesis and characterization of oil-soluble Ag NPs and MIPs, the selectivity of MIPs for PCNB, and the limit of detection (LOD) of PCNB as well as the corresponding linearity range were investigated in this work, which, for the first time, provides a potential point-of-care tool for detecting PCNB residue in food with high sensitivity and selectivity by employing SERS-MIPs technology.

## 2. Materials and Methods

### 2.1. Instruments and Reagents

The morphology of colloidal silver particles and Ag NPs-embedded MIPs were observed on a transmission electron microscope (TEM, Tecnai G2 F30 S-Twin, FEI Company, Hillsboro, OR, Netherlands) operating at an acceleration voltage of 300 kV. The TEM was equipped with an energy dispersive X-ray (EDX) analyzer (DPP-II). The size of the Ag NPs was measured using the Nano measure 1.2, and further automatically counted through the Image J. SERS spectra were collected on a DeltaNu 785 Raman spectrometer (DeltaNu Inc., Laramie, WY, USA). The laser power of the spectrometer is 120 mW with an excitation wavelength at 785 nm and a spectral range of 200 to 2000 cm^−1^. The spectra were acquired with baseline off using NuSpec software (Copyright DeltaNu 2009) and analyzed using GRAMS/AI software (Ver 9.1, Thermo Fischer Scientific, Waltham, MA, USA).

X-ray diffraction (XRD) patterns were recorded with a PANalytical X’Pert PRO powder diffractometer (X-ray diffractometer, PANAlytical, the Netherlands) using Cu Kα radiation (λ = 0.1541 nm). The working voltage was 40 kV and the working current was 40 mA. The patterns were collected with a 2θ range from 0° to 90° at a step of 0.0167°.

Trifluoroacetic acid silver salt (98%), triethylsilane (99%), PCNB (95%), oleylamine (85–90%) and oleic acid (AR) were purchased from Shanghai Aladdin Bio-Chem Technology Co., Ltd. (Shanghai, China). Tetrahydrofuran (AR) and chloroform (AR) were bought from Shanghai Lingfeng Chemical Reagent Co., Ltd. (Shanghai, China). Methylbenzene was obtained from Hangzhou Shuanglin Chemical Reagent Co., Ltd. (Zhejiang, China). Methyl methacrylate (AR),1,4-butanediodethacrylate (95%), LPO (AR) and 4-nitrochlorobenzene (99.5%) were purchased from Shanghai Aladdin Bi Co., Ltd. (Shanghai, China). 3,5-Dichloronitrobenzene (98%) purchased from Shanghai Bide Pharmaceutical Technology Co., Ltd. (Shanghai, China). 1,2,3- Trichloro-5-nitrobenzene (98%) were from Shanghai McLean Biochemistry Co., Ltd. (Shanghai, China).

### 2.2. Synthesis of Oil-Soluble Silver Nanoparticles

Prior to experiments, all glasswares were bathed in freshly prepared aqua regia (*v*/*v* HNO_3_:HCl = 1:3) for 4 h, then rinsed thoroughly with distilled water, and soaked in an alkali tank (sodium hydroxide ethanol solution, 50 g/L) for 4 h. Finally, the glassware should be rinsed with ultra-pure water and put into an electric thermostatic drying oven.

Briefly, 10.0 mg of silver trifluoroacetic acid was measured in a 5 mL centrifuge tube and 1.0 mL of tetrahydrofuran was added. The solution was ultrasonicated for 1 min until the silver trifluoroacetic acid was completely dissolved. The dissolved solution was transferred to the reaction bottle, and then 9.0 mL of tetrahydrofuran, 3.0 μL of oleoamine and 15.0 μL of oleic acid were successively added into the reaction bottle.

Subsequently, the reaction bottle was placed on the digital display constant temperature heating plate at 50 ℃, heated for 10 min to the constant temperature, and then 10.0 μL of triethylsilane was added into the solution. At this time, the solution changed from colorless to dark brown rapidly, with continued heating at constant temperature for 60 h. When the reaction was completed, the heating and stirring were stopped and the color of the solution changed from dark brown to reddish brown. Finally, the prepared oil-soluble silver nanoparticles sol was cooled to room temperature and stored in a refrigerator at 4 ℃.

### 2.3. Preparation of Molecularly Imprinted Polymers (MIPS) and Non-Printed Polymers (NIPS) Containing Oil-Soluble Silver Nanoparticles

A typical process for the synthesis of MIPs containing oil-soluble silver nanoparticles is as follows.

First, the prepared nano-sol was mixed with equal volume of methanol and centrifuged to concentrate silver nanoparticles. The MMA monomer was purified using an alkaline alumina column to remove the inhibitor and the remaining oil-soluble silver nanoparticles were ultrasonically dispersed in the purified MMA.

A magnetic agitator was added to 5.0 mL reaction tube, followed by the addition of 1.0 mL of MMA monomer, then 0.05 mL of 1,4-butanediol dimethacrylate crosslinking agent and 20.0 mg of LPO initiator were added successively.

Afterwards, 20.0 mg PCNB was added to the mixture which was kept in an ultrasonic bath for 3 min to achieve complete homogenization. Then, the solution was degassed with nitrogen gas for 10 min. After that, the reaction tube was put into the thermostatic heating plate at a constant temperature of 60 °C, with continued stirring at a speed of 300 r/min. After about 40 min, the polymerization was stopped and the reaction tube was cooled to room temperature.

After the polymerization was completed, the polymer was taken out and crushed (650 W, 25,000 r/min) into fine particles, and then ground in a grinding bowl. The imprinted polymer powder was collected through a 100-mesh steel sieve, wrapped in a filter paper, transferred to the soxhlet extractor, and the PCNB molecules were removed by toluene elution for 8 h until the PCNB could not be detected in the MIPs by SERS. The MIPs powder with PCNB completely removed was collected and placed in a dryer for later use.

By comparison, oil-soluble Ag NPs containing non-imprinted polymers (NIPs) were synthesized according to the same procedure in the absence of PCNB. All polymers were vacuum dried overnight before they were used in re-binding studies.

### 2.4. Detection of PCNB Using Oil-Soluble Ag NPs-Embedded MIPs as a Substrate

The kinetic adsorption tests were carried out to determine the binding equilibrium time of MIPs and NIPs. First, nine samples of 5.0 mg of oil-soluble Ag NPs-embedded MIPs and NIPs were mixed with 2 mL of PCNB solution at a concentration of 1.0 μg/mL. Then the mixtures were incubated under shaking for various time intervals. Between 30 min and 150 min, the centrifuge tubes equipped with MIPs and NIPs was taken out at 15 min intervals and centrifuged at 8000 r/min for 10 min. The precipitated polymer was washed three times with toluene to remove PCNB on its surface. After natural drying, 10 different positions of the polymer were selected and interrogated by laser with integration time of 10 s. Each experiment was repeated three times to obtain the average value. The intensity of 1600 cm^−1^ peak corresponding to the stretch vibration of benzene ring in PCNB was selected to plot against the adsorption time.

For the re-binding studies of PCNB, 5.0 mg of oil-soluble Ag NPs-embedded MIPs were mixed with 2.0 mL of PCNB toluene solution at different concentrations (0.15 μg/mL, 0.1 μg/mL, 0.05μg/mL, 0.01 μg/mL, 0.005 μg/mL), and the mixture was incubated at room temperature under shaking for 3 h. Then, the mixture was centrifuged at 8000 rpm for 10 min and the supernatant was removed. The precipitated polymers were washed with steamed toluene and separated by centrifugation. Then naturally dried polymers was observed under the microscope of Raman spectrometer. The spectrum was recorded, and each spectrum was an average of 10 independent results taken at an integration time of 10 s. The logarithmic intensity of the characteristic peak of PCNB at 1600 cm^−1^ was used to plot against the logarithmic concentration of PCNB to build a calibration curve. The detection of PCNB using oil-soluble Ag NPs-embedded NIPs as a substrate was performed according to the same procedure described above (except for the fact that the MIPs were replaced with NIPs).

### 2.5. Application of the Oil-Soluble Ag NPs-Embedded MIPs in Food Samples

The rice sample (Hangzhou Rice Technology Co., Ltd., Hangzhou, China) used in this work was purchased from a local supermarket in Hangzhou, Zhejiang, China. PCNB was not detected in the origin sample. The rice sample was taken out and crushed (650 W, 25,000 r/min) into fine particles, and then ground in a grinding bowl. The rice sample powder was collected through a 100-mesh steel sieve. A series of aliquot samples (5.0 mg) were spiked with PCNB at concentrations of 0.12 μg/mL, 0.06 μg/mL, 0.03 μg/mL, respectively, for soaking until the solvent dried naturally and PCNB adhered to the surface of the rice sample. Then, the PCNB-spiked samples were handled and determined by the procedures described in Section 2.4. Recoveries were calculated and three parallel analyses were performed for each rice sample. The data were expressed as the mean ± standard deviation (SD).

### 2.6. Determination of PCNB in Rice by GC-MS

In this section, the method stipulated in GB/T23200.113-2018 [33] was used to analyze PCNB in rice by gas chromatography-mass spectrometry (GC-MS), and the detection results were compared with SERS-MIPs method in order to evaluate the reliability of detecting PCNB in rice employing SERS-MIPs technology. Standard solutions of PCNB with concentration gradients of 0.15 μg/mL, 0.10 μg/mL, 0.05 μg/mL, 0.01 μg/mL and 0.005 μg/mL were prepared and injected into GC-MS chromatograph for analysis. The concentration of PCNB was plotted logarithmically against the peak area of PCNB in the chromatography, and the calibration curve and correlation coefficient were obtained after fitting.

The spiked process of rice is the same as the procedures described in Section 2.5. After the spiked rice was crushed and screened, n-hexane was used for extraction, and the extracted liquid was purified by silica and magnesium adsorbent pretreatment column, and then concentrated by nitrogen blowing, filtered by 0.22 μm microporous filter membrane. Under the chromatographic conditions specified by national standard, automatic sampling analysis was carried out by GC-MS analyzer.

The analytical conditions of GC-MS were as follows: HP-5 MS column (30 m × 0.25 mm × 0.25 μm), inlet temperature 280 ℃, detector temperature 300 °C. Carrier gas and tail gas were nitrogen with high purity (purity >99.99%), column flow rate was 1.0 mL/min, sample volume was 1.0 μL, and the injection method was splitless injection. The ionization mode is Ei, and the ionization energy is 70 eV. The ion source temperature is 280 °C; The temperature of the transmission line was 280 ℃, and the solvent delay was 3 min. The GC oven temperature was raised to 40 °C at the rate of 40 °C/min, then raised to 120 °C at the rate of 5 °C/min, and then raised to 240 ℃ at the rate of 40 °C/min at the rate of 40 °C/min, and held for 1 min respectively. Finally, after the temperature was raised to 300 °C, it was kept for 6 min and then the heating procedure was stopped. (Table S1).

## 3. Results and Discussion

### 3.1. Characterization of the Oil-Soluble Ag NPs

**Figure 2:**
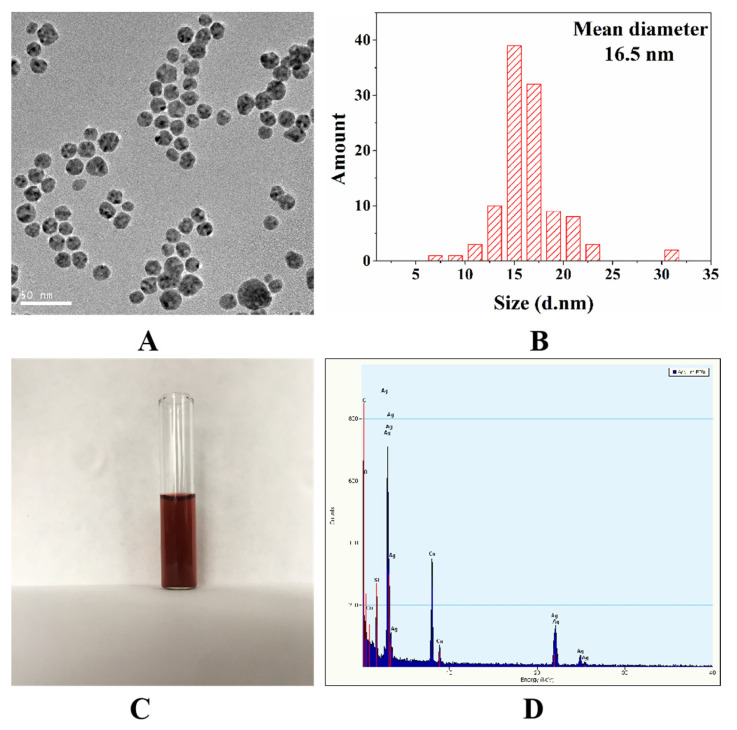
TEM image (**A**), size (**B**), picture (**C**) of the oil-soluble Ag NPs; energy dispersive X-ray (EDX) spectrum (**D**) of the oil-soluble Ag NPs.

Noble metal colloids are extensively employed as SERS substrates due to their easy preparation, stable performance, and high sensitivity. Generally, Au nanoparticles (Au NPs) have better chemical stability than Ag nanoparticles (Ag NPs). However, Ag NPs possess stronger SERS activity than Au NPs, which makes it a desirable SERS substrate [27]. Figure 2A shows the TEM image of oil-soluble Ag NPs at 50 nm scale. Obviously, the size of silver nanoparticles is uniform, the particles are arranged closely and neatly, and there is a gap of uniform size between particles, which is conducive to the generation of “hot spot effect”. The average number diameter of silver nanoparticles measured by Nanomeasure 1.2 is 15.8 ± 3.9 nm. Figure 2B demonstrates that the hydrodynamic diameter of the oil-soluble Ag NPs detected by DLS was monomodal with an average size of 16.5 ± 3.1 nm, which was comparable to the average diameter of Ag NPs measured by TEM. Figure 2C are photographs of the corresponding silver nanoparticle solvent, indicating that the silver solvent color is reddish brown. Figure 2D shows the EDX spectrum of the Ag NPs displays Ag signal peaks from Ag nanoparticles and Cu peaks as well as C peaks from the ultrathin carbon-coated copper grid.

### 3.2. Characterization of the Oil-Soluble Ag NPs in MIPs

**Figure 3:**
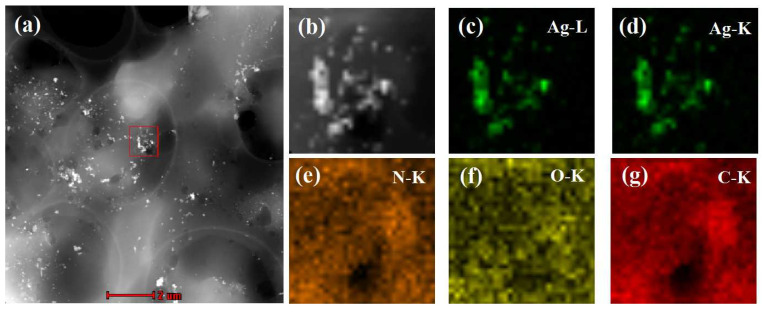
High-angle annular dark-field scanning transmission electron microscopy (HAADF-STEM) of MIPs containing silver nanoparticles images (**a**,**b**) and compositional maps of silver (**c**,**d**), nitrogen (**e**), oxygen (**f**), and carbon (**g**).

Dry MIPs were ground into fine powders, ultrasonically dispersed in tetrahydrofuran (THF) at room temperature, and transferred onto a carbon-coated copper grid by dipping. The elemental distribution of MIPs was then measured by EDX mapping technique in high-angle annular dark-field scanning transmission electron microscopy (HAADF-STEM) mode. In a selected area (outlined in red in Figure 3a,b), the corresponding element mapping of silver (Figure 3c,d) from oil-soluble Ag NPs and the mapping of nitrogen (Figure 3e), oxygen (Figure 3f), and carbon (Figure 3g) from molecular imprinted polymers clearly indicate that there were large quantities of silver nanoparticles distributed in the MIPs. Therefore, the MIPs could be used as an SERS active substrate for the detection of PCNB.

Dry MIP powder was detected using a PANalytical X’Pert PRO powder diffractometer. Figure S1 showed an XRD profile of the Ag NPs-embedded MIPs, and the resulting XRD data were processed by the JADE software. In the figure, the peaks of the silver located at 2θ = 38.081, 44.279, 64.440, 77.438, and 81.597 correspond to the (1 1 1), (2 0 0), (2 2 0), (3 1 1), and (2 2 2) crystal faces of the silver nanoparticles, respectively. The average crystallite sizes of the samples were calculated with the Scherrer equation based on the strongest hkl (2 2 0) diffraction peak of Ag, it was concluded that the crystalline grain size of this silver nanoparticle was 23.8 nm.

### 3.3. SERS Selectivity of the Oil-Soluble Ag NPs-Embedded MIPs

A large number of functional groups and blotted holes are provided by MIPs to specifically identify PCNB molecules and to apply their specific adsorption to the surface of oil-soluble silver nanoparticles. The adsorption time affects MIPs’ recognition and rebinding of PCNB. If the adsorption time is too short, MIPs cannot completely identify and fully combine with PCNB in the environment. If the adsorption time is too long, the experimental efficiency will be greatly reduced. Therefore, it is necessary to determine the optimal equilibrium time. The kinetic adsorption test (Figure S2) showed that MIPs had a significant stronger adsorption capacity for PCNB than NIPs, mainly due to the fact that NIPs could only interact with PCNB molecules through physical adsorption, so PCNB molecules could not be specifically adsorbed by NIPs. It could also be seen from the figure that when the adsorption time was between 30–120 min, the peak intensity of PCNB at 1600 cm^−1^ increased significantly in MIP group. After 120 min, there was no distinct change in the peak intensity, indicating that the adsorption of PCNB by MIPs reached saturation and that 120 min is the optimal equilibrium time.

**Figure 4:**
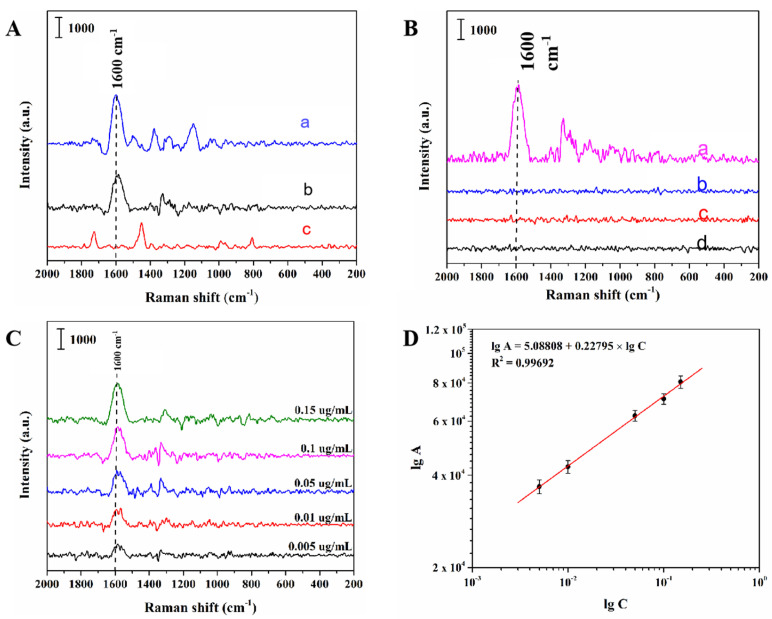
(**A**) Surface-enhanced Raman spectroscopy (SERS) spectra of PCNB with oil-soluble silver nanometer as substrate (a), MIPs absorbing 1 µg/mL of PCNB (b), and NIPs absorbing 1 µg/mL of PCNB. (**B**) The SERS spectra acquired using the oil-soluble Ag NPs-embedded MIPs as substrates after the MIPs were respectively incubated with the solutions of PCNB (a), 3, 4, 5-trichloronitrobenzene (b), 3, 5-dichloronitrobenzene(c), and para-chloronitrobenzene (d) for 120 min at the concentration of 1.0 µg/mL. (**C**) SERS spectra of PCNB using the MIP substrate incubated with solutions of PCNB at different concentrations. (**D**) Linear correlation of the logarithmic peak intensity at 1600 cm^−1^ versus the logarithmic concentrations of PCNB.

Then, in order to further validate the selectivity of MIPs prepared for PCNB in this study, MIPs and NIPs were incubated with PCNB solution of 1.0 μg/mL followed by SERS interrogation, and the obtained experimental results are shown in Figure 4A. Spectrum a shows the surface-enhanced Raman spectrum of PCNB with oil-soluble Ag NPs as a substrate. Spectrum b presents the spectrum of PCNB detected with MIPs, and the distinguishing characteristic peak of PCNB could be observed at 1600 cm^−1^ which correspond to the stretch vibration of benzene ring in PCNB. For NIPs, SERS detection was conducted under the same conditions as shown in spectrum c, but no characteristic peak of PCNB was appeared. These results illustrated that the oil-soluble Ag NPs-embedded MIPs had high selectivity and affinity for PCNB due to the specificity of the cavities in the imprinted template.

### 3.4. SERS Specificity of the Oil-Soluble Ag NPs-Embedded MIPs

PCNB and its structural analogues (p-chloronitrobenzene, 3, 5-dichloronitrobenzene and 3, 4, 5-trichloronitrobenzene) were selected for evaluating the assay specificity, in other words, to verify the ability of MIPs to specifically absorb PCNB from a complex matrix. Briefly, 5.0 mg MIPs was added to 2.0 mL, 1.0 μg/mL of PCNB, p-chloronitrobenzene, 3,5-dichloridrobenzene, and 3,4,5-trichloronidrobenzene solutions respectively, and was incubated for 120 min. Then the MIPs were used as SERS substrates to measure spectrum of each analogue.

As can be seen from Figure 4B, the SERS spectra of MIPs adsorbed by PCNB solution showed distinct characteristic peak signals at 1600 cm^−1^, while in contrast, the spectra of MIPs adsorbed by p-chloronitrobenzene, 3, 5-dichloronitrobenzene and 3, 4, 5-trichloronitrobenzene solution did not show characteristic peaks of corresponding substances. The experimental results showed that the MIPs prepared by this method could produce cavities with molecular imprinted PCNB after elution, and the binding sites in the cavities could match the functional groups of PCNB and their spatial arrangement order. Even if the analogues were dispersed into the cavities, they could not be completely combined with the binding sites. Therefore, MIPs prepared by this method has the capability to specifically detect PCNB from complex matrix.

### 3.5. Detection of PCNB Spiked Using Oil-Soluble Ag NPs-Embedded MIPs as a Substrate

Under the optimal experimental conditions, standard solution of PCNB with a concentration of 0.005–0.15 μg/mL was interrogated by laser to obtain the calibration curve. Figure 4C shows the SERS spectra of PCNB solutions detected at different concentrations. A progressive decrease in peak area intensity at 1600 cm^−1^ was displayed when the PCNB concentration decreased from 0.15 to 0.005 µg/mL. As shown in Figure 4D, within the concentration range of 0.005–0.15 μg/mL, the logarithm (lgC) of PCNB solution concentration had a good linear relationship with the logarithm (lgA) of Raman peak area at 1600 cm^−1^. The linear equation was lgA = 5.08808 + 0.22795 × lgC (R^2^ = 0.9969). On the basis of the judgment standard of the minimum detection limit [34], when the ratio of signal intensity (S) to noise intensity (N) of the tested sample is greater than or equal to 3, it is considered to be an effective signal. Therefore, the minimum detection limit of this method is 0.005 μg/mL.

According to the experimental data (Table S2), the recoveries and RSD of PCNB were 95.0% to 102.7% and 3.9% to 6.1%, respectively. The feasibility of combining MIPs with SERS technology was proved through experiment, and the utility of detecting PCNB residues in food with SERS-MIPs was also confirmed.

### 3.6. Detection of PCNB Spiked in a Rice Using the MIPs as a Substrate

The MIPs were further applied to detect PCNB spiked in rice (Hangzhou Rice Technology Co., Ltd., Hangzhou, China). The results indicated that the recoveries of samples were found to be in the range of 94.4% to 103.3% and the values of RSD were between 4.6% to 7.4% (Table S3, Figure S3). The good recovery and RSD indicated that the oil-soluble Ag NPs-embedded MIPs were suitable for the detection of PCNB in real samples.

In addition, the pretreatment of samples is not complicated. Only a small amount of sample crushing and centrifugation are required, which can simplify the experimental processing steps and save the detection cost.

### 3.7. Determination of PCNB in Rice by GC-MS

In order to further verify the accuracy of the experimental results of detecting PCNB in food matrix with MIPs as SERS substrate, according to the method specified in GB/T23200.113-2018, this section uses GC-MS to carry out quantitative analysis of PCNB in rice sample spiked with PCNB. The detection results were compared with those by SERS-MIPs. Figure S4A shows the chromatogram of 20.0 μg/mL PCNB solution measured by GC-MS. The retention time of PCNB was 12.19 min.

Figure S4B demonstrates a linear plot of the logarithmic of peak area versus the logarithm of the PCNB concentration, indicating that the detection is linear within the concentration range from 0.005 to 0.15 µg/mL. The linear equation was lgA = 8.52006 + 0.97061× lgC (lgA refers to the logarithmic peak area, lgC refers to the logarithmic concentration of PCNB) and the coefficient of this regression was determined to be R^2^ = 0.99499.

The experimental results are shown that the recoveries were found to be 94.8% to 96.7% and the values of RSD were in the range of 0.97% to 1.39% (Table S4, Figure S5). The GC-MS results are consistent with that by SERS-MIPs method, indicating that the rapid detection of PCNB in food matrix by SERS combined with MIPs is reliable.

This method was compared with other commonly used methods for detecting PCNB in plant foods, and the results were shown in Table 1. It can be seen from the table that the detection linear range of SERS-MIPs method was wider than that of Auto-SPE GC-MS [35], HPLC [36] and Quechers-GC [37]. The SERS-MIPs method was simpler than the matrix solid phase dispersion extraction-gas chromatography (MSPD-GC) method [38].

Overall, the proposed method is fast in detection without tedious sample pretreatment. Moreover, it can specifically recognize and adsorb PCNB in complex food matrices. In addition, this method only requires small amount of samples in the detection process, which can save the detection cost. The portable Raman spectrometer can also realize the rapid detection in an outdoor field.

## 4. Conclusions

In this study, the prepared MIPs, which was embedded with oil-soluble silver nanoparticles as SERS substrate, can specifically recognize PCNB, providing a sensitive, selective, onsite, and rapid detection method for PCNB residue in food by SERS-MIPs. Due to the insolubility of PCNB in water, a special type of oil-soluble silver nanoparticle was synthesized, which can be expanded to detect oil-soluble toxic substances. By using oleamine and oleic acid as protective agents, the silver nanoparticles were soluble in organic phase so that they could contact with PCNB molecules. Then, SERS was employed to realize the detection of PCNB.

The linear range was 0.005 to 0.15 µg/mL and the LOD was 0.005 μg/mL for the detection of PCNB spiked in water, and the corresponding recoveries ranged from 95.0% to 102.7% with RSD in the range of 3.9% to 6.1%. The RSD and recovery rates for the detection of PCNB spiked in a rice sample (Hangzhou Rice Technology Co., Ltd., Hangzhou, China) were 4.6% to 7.4% and 94.4% to 103.3%, respectively. Quantitative analysis of PCNB spiked in the rice samples by GC-MS showed the recoveries were from 94.8% to 96.7% and the values of RSD were in the range of 0.97% to 1.39%. As a whole, the proposed method provides a potential point-of-care and cost-effective tool for rapidly detecting pesticide residue in food matrices with high sensitivity and selectivity.

## Figures and Tables

**Table 1:** Comparison of SERS-MIPs methods with other reported methods.

Method	Linear Range	LOD (mg/kg)	Recovery (%)	RSD (%)
Auto-SPE GC-MS	0.1–2.0 mg/L	0.001	83.6–96.5	5.5–7.1
HPLC	0.1–50 mg/L	0.02	83.2–94.2	Less than 5.5
MSPD-GC	0.005–0.5 μg/mL	0.002	89.4–102.4	3.7–7.4
QuEChERS-GC	0.02–0.3 mg/kg	0.002	86.3–105.4	Less than 4.15
SERS-MIPs	0.005–0.15 μg/mL	0.005	94.4–103.3	4.6–7.4

## Data Availability

Raw data presented in this study are available on request from the corresponding author.

## Supplementary Materials

The following are available online at https://www.mdpi.com/article/10.3390/bios12020052/s1, Figure S1: XRD pattern of the Ag NPs-embedded MIPs, Figure S2: Kinetic adsorption test of MIPs versus NIPs, Figure S3: Spectra of PCNB in rice detected by SERS-MIPs method, Figure S4: (A) GC-MS chromatogram of 20.0 μg/mL PCNB solution. (B) Standard curve for detecting PCNB in rice by GC-MS, Figure S5: The chromatogram of PCNB in rice measured by GC-MS, Table S1: Temperature program of the GC-MS column, Table S2: Percentage recoveries and relative standard deviation (RSD) of the detection of PCNB spiked in toluene samples (mean ± SD, N = 3), Table S3: Recovery and precision analysis of detection of spiked PCNB in rice using MIPs as SERS substrates, Table S4: Recovery and precision analysis of detection of PCNB spiked in rice using GC-MS.
