# Supplementary material for: Rapid and Sensitive Detection of Pentachloronitrobenzene by Surface-Enhanced Raman Spectroscopy Combined with Molecularly Imprinted Polymers
Source: Biosensors (Basel). 2022 Jan 19;12(2):52. doi: 10.3390/bios12020052 (PMC8869095; doi:10.3390/bios12020052)
Supplement: Supplementary file 1 [file biosensors-12-00052-s001.zip › biosensors-1529188-supplementary.pdf]

biosensors

Supplementary material: Rapid and Sensitive Detection of Pentachloronitrobenzene by Surface-Enhanced Raman Spectroscopy Combined with Molecularly Imprinted Polymers

Table S1. Temperature program of the GC-MS column.

Procedure	Temperature (°C)	Time (min)	Heating rate (℃/min)
1	40	1	40
2	120	1	5
3	240	1	12
4	300	6	

In section 2.6, the heating procedure of chromatographic column is shown in Table S1.

Table S2. Percentage recoveries and relative standard deviation (RSD) of the detection of PCNB spiked in toluene samples (mean ± SD, N = 3).

Concentration of PCNB solution （μg/mL）	Found ± SD（μg/mL）	Recovery（%）	RSD（%）
0.130	0.128 ± 0.078	98.5	6.1
0.080	0.076± 0.032	95.0	4.3
0.040	0.030 ± 0.016	102.7	3.9

In section 3.5, Table S2 summarizes the recovery and the relative standard deviation (RSD) of the detection of PCNB.

Table S3. Recovery and precision analysis of detection of spiked PCNB in rice using MIPs as SERS substrates.

Rice Sample	Spiked（μg/mL）	Found ± SD（μg/mL）	Recovery（%）	RSD（%）
1	0.120	0.113 ± 0.052	94.4	4.6
2	0.060	0.059± 0.027	97.5	4.7
3	0.030	0.031 ± 0.023	103.3	7.4

In section 3.6, The MIPs were further applied to detect PCNB spiked in rice (Hangzhou Rice Technology Co., Ltd.). As shown the recoveries of samples and the values of RSD were found in Table S3.

Table S4. Recovery and precision analysis of detection of PCNB spiked in rice using GC-MS.

Rice Sample	Spiked（μg/mL）	Found ± SD（μg/mL）	Recovery（%）	RSD（%）
1	0.120	0.114 ± 0.001	94.8	0.97
2	0.060	0.057 ± 0.0008	95.8	1.39
3	0.030	0.290 ± 0.0003	96.7	1.03

In section 3.7, the recoveries of samples and the values of RSD were demonstrated in Table S4.

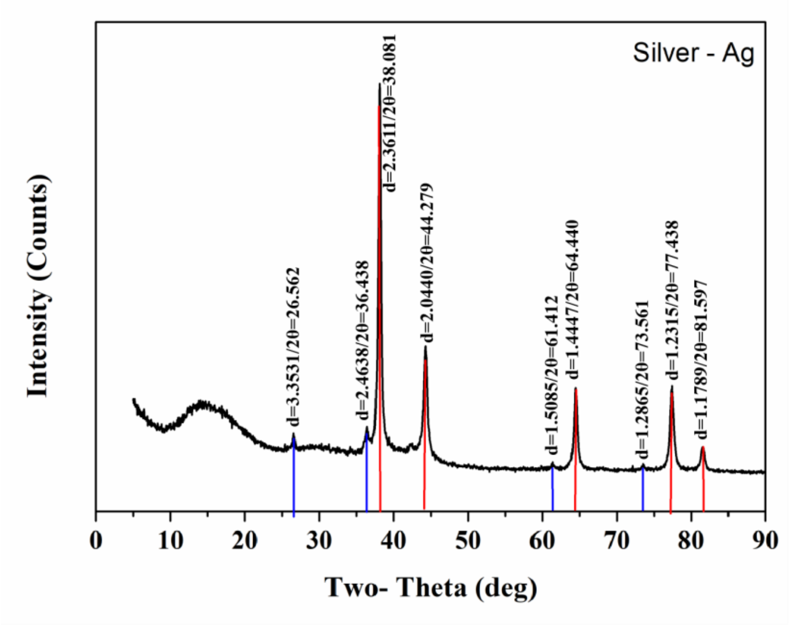

Figure S1. XRD pattern of the Ag NPs-embedded MIPs.

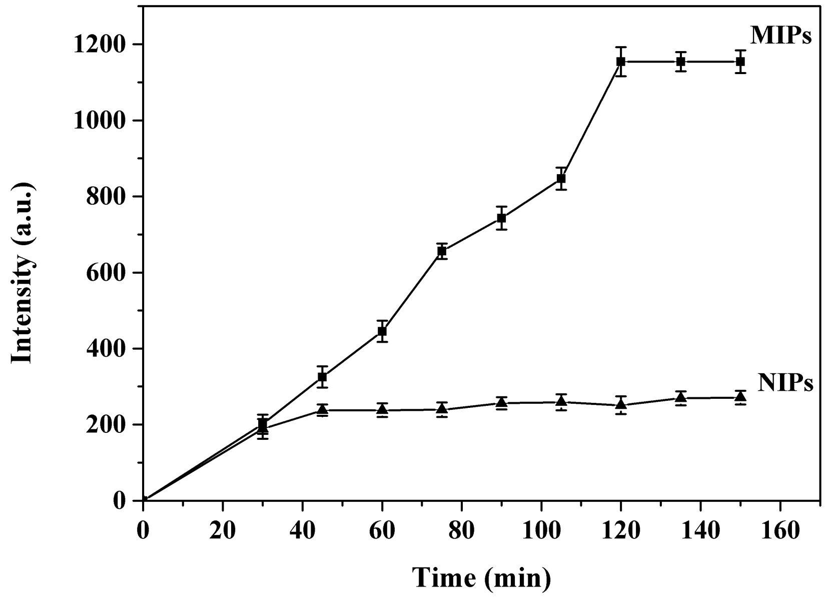

Figure S2. Kinetic adsorption test of MIPs versus NIPs. The characteristic PCNB peak intensity at 1600 cm-1 was plot against the adsorption time. Initial concentration of PCNB was 1.0 μg/mL. Time points were 30, 45, 60, 75, 90, 105, 120, 135, 150 min.

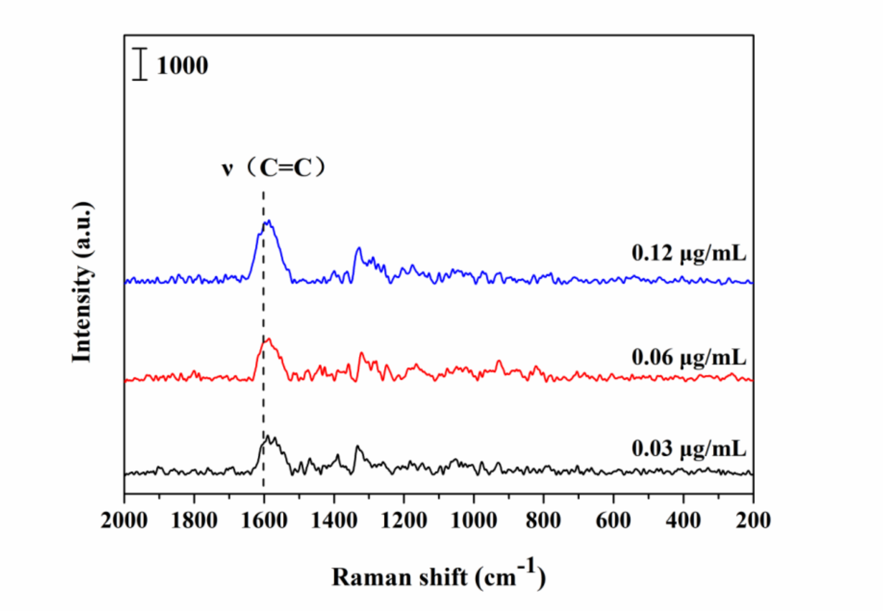

Figure S3. Spectra of PCNB in rice detected by SERS-MIPs method.

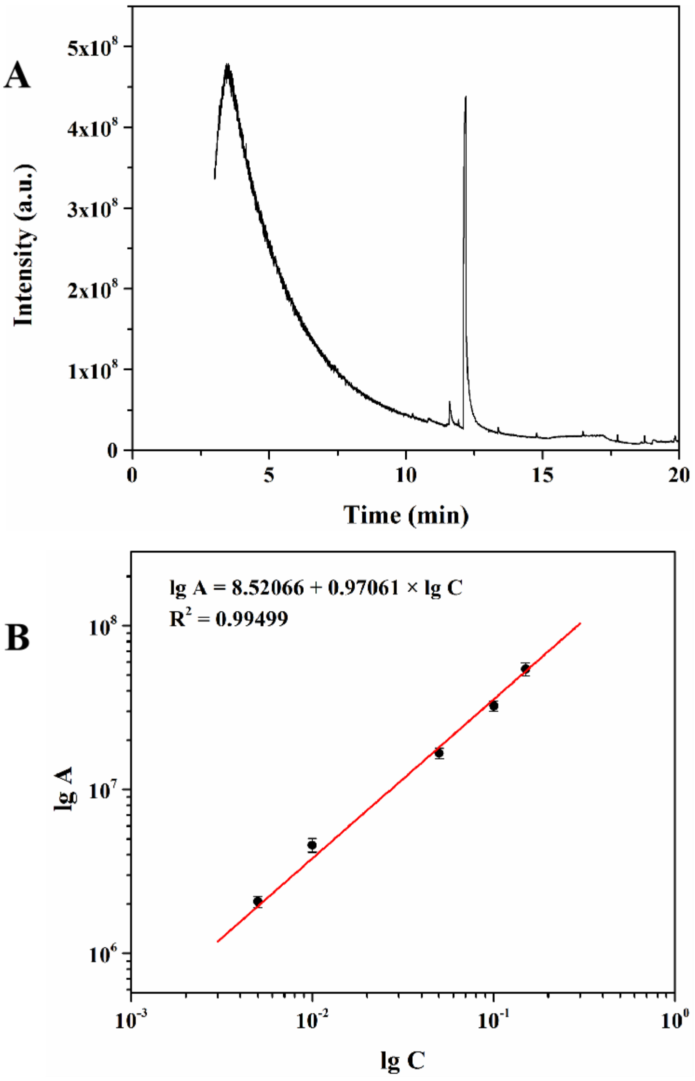

Figure S4. (**A**) GC-MS chromatogram of 20.0 μg/mL PCNB solution. (**B**) Standard curve for detecting PCNB in rice by GC-MS.

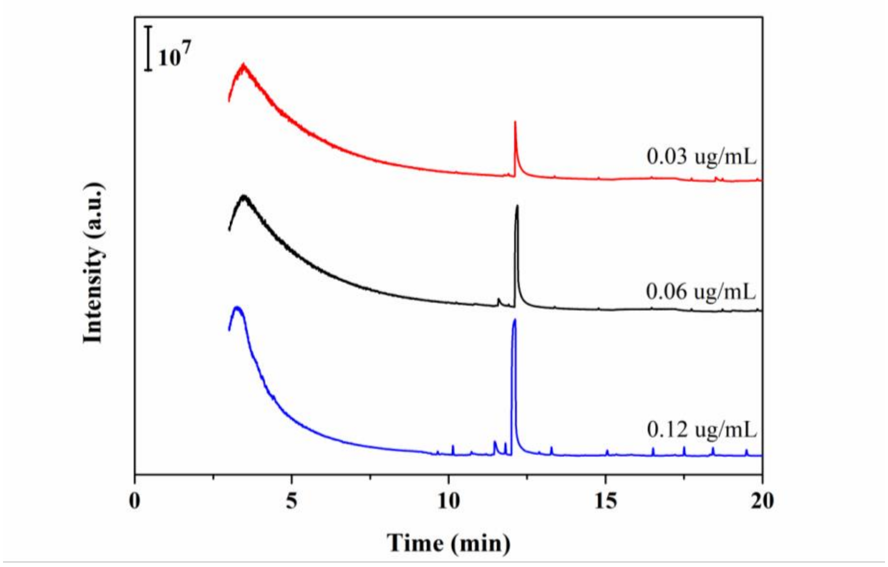

Figure S5. The chromatogram of PCNB in rice measured by GC-MS.
